# Addressing head motion dependencies for small-world topologies in functional connectomics

**DOI:** 10.3389/fnhum.2013.00910

**Published:** 2013-12-26

**Authors:** Chao-Gan Yan, R. Cameron Craddock, Yong He, Michael P. Milham

**Affiliations:** ^1^Nathan Kline Institute for Psychiatric ResearchOrangeburg, NY, USA; ^2^Center for the Developing Brain, Child Mind InstituteNew York, NY, USA; ^3^The Phyllis Green and Randolph Cowen Institute for Pediatric Neuroscience, New York University Child Study CenterNew York, NY, USA; ^4^State Key Laboratory of Cognitive Neuroscience and Learning & IDG/McGovern Institute for Brain Research, Beijing Normal UniversityBeijing, China; ^5^Center for Collaboration and Innovation in Brain and Learning Sciences, Beijing Normal UniversityBeijing, China

**Keywords:** functional connectomics, head motion impact, network analysis, resting-state fMRI, small-world, topological parameters

## Abstract

Graph theoretical explorations of functional interactions within the human connectome, are rapidly advancing our understanding of brain architecture. In particular, global and regional topological parameters are increasingly being employed to quantify and characterize inter-individual differences in human brain function. Head motion remains a significant concern in the accurate determination of resting-state fMRI based assessments of the connectome, including those based on graph theoretical analysis (e.g., motion can increase local efficiency, while decreasing global efficiency and small-worldness). This study provides a comprehensive examination of motion correction strategies on the relationship between motion and commonly used topological parameters. At the individual-level, we evaluated different models of head motion regression and scrubbing, as well as the potential benefits of using partial correlation (estimated via graphical lasso) instead of full correlation. At the group-level, we investigated the utility of regression of motion and mean intrinsic functional connectivity before topological parameters calculation and/or after. Consistent with prior findings, none of the explicit motion-correction approaches at individual-level were able to remove motion relationships for topological parameters. Global signal regression (GSR) emerged as an effective means of mitigating relationships between motion and topological parameters; though at the risk of altering the connectivity structure and topological hub distributions when higher density graphs are employed (e.g., >6%). Group-level analysis correction for motion was once again found to be a crucial step. Finally, similar to recent work, we found a constellation of findings suggestive of the possibility that some of the motion-relationships detected may reflect neural or trait signatures of motion, rather than simply motion-induced artifact.

## Introduction

The graph of functional interactions in the human connectome is increasingly being used as a defining component of an individual's neurophenotype (Craddock et al., 2013). Not surprisingly, cataloging variations in the connectome, from one individual or population to another, has emerged as a key objective in modern day neuroscience. Seemingly simple from a conceptual viewpoint, the task of characterizing and comparing connectomes has proven to be a significant challenge for the imaging community—both due to the computational complexity of the connectome graph and the richness of interactions between its connections and subgraphs (i.e., modules). In response, the examination of connectomes in terms of their network properties has emerged as a potentially promising solution that reduces its complexity to a set of topological parameters (see Table 1) that are easily amenable to comparison across individuals and populations (Bullmore and Sporns, 2009). Initial studies have demonstrated the sensitivity of these measures to differences in both diagnostic status and behavioral indices (Bassett and Bullmore, 2009; Bullmore and Sporns, 2009, 2012; He and Evans, 2010; Wang et al., 2010; Bullmore and Bassett, 2011; Yu et al., 2012), and have exhibited acceptable test-retest reliability for these metrics (Telesford et al., 2010; Wang et al., 2011). Although promising, little attention has been given to the potential confounding effects of nuisance signals present in R-fMRI studies—in particular, that of motion, which is the primary focus of the present work.

**Table 1:** **Topological properties of brain graphs examined in the current study**.

**Topological properties**	**Descriptions**
**GLOBAL TOPOLOGICAL PROPERTIES**	
Local efficiency	The average efficiency of information transfer over a node's direct neighbors
Global efficiency	The efficiency of information transfer through the entire graph
Clustering coefficient	The average inter-connectedness of a node's direct neighbors
Characteristic shortest path length	The average shortest path length between any pairs of nodes
Normalized clustering coefficient	The clustering coefficient compared to matched random networks
Normalized characteristic shortest path length	The characteristic shortest path length compared to matched random networks
Small-worldness	The normalized clustering coefficient divided by the normalized characteristic shortest path length, which reflect the balance of global efficiency and local efficiency
Assortativity	The tendency of nodes to link with those nodes with similar number of edges
Modularity	The extent to which a graph can be segregated into densely intraconnected but sparsely interconnected modules
**REGIONAL TOPOLOGICAL PROPERTIES**	
Degree centrality	The number (or sum of weights) of connections connected directly to a node
Nodal efficiency	The efficiency of information transfer over a node's direct neighbors
Nodal clustering coefficient	The inter-connectedness of a node's direct neighbors
Subgraph centrality	The participation of a node in all subgraphs comprised in a graph
Betweenness centrality	The influences of a node over information flow between other nodes
Eigenvector centrality	A self-referential measure of centrality – nodes have high eigenvector centrality if they connect to other nodes that have high eigenvector centrality

Although the impacts of motion on graph topological measures have not been thoroughly assessed, the demonstrated deleterious effects of motion on community detection provides compelling evidence of their existence (Power et al., 2012). Previous work has found that the assignment of nodes in the connectome to communities (modules) differed notably between children and adults when motion was not considered, but were more similar when motion was accounted for by the removal of affected frames (i.e., scrubbing) (Power et al., 2012). Beyond this demonstration, a key point raised by Power et al., as well as others (Satterthwaite et al., 2012; Van Dijk et al., 2012) is that short-distance connectivity increases with motion while long-distance connectivity decreases. However, applying this knowledge to topological parameters does not lead to any direct conclusions. In topological space nodes are deemed neighbors if they are directly connected, regardless of the anatomical distance between them. Thus, although motion may decrease the number of long-distance connections in a node's topological neighborhood, and increase the number of short-distance connections, the overall impact to topological parameters such as local and global efficiency is unclear. Equally unclear is the degree to which the effects of motion are reflected in compromises to small-world properties, which reflect the balance of global efficiency with local efficiency (Watts and Strogatz, 1998; Salvador et al., 2005).

Concerns about the impact of motion on topologic parameters are particularly relevant to studies of inter-individual or population-based differences, where systematic relationships can exist between motion and variables of interest (e.g., developmental status, diagnostic status).

In this regard, several recent studies focusing on seed-based correlation and regional R-fMRI measures (Fair et al., 2012; Satterthwaite et al., 2013; Yan et al., 2013a; Power et al., 2014) have provided comprehensive assessments of motion related artifacts and suggested measures for controlling them. These studies generally emphasize that, if attempting to correct for motion at the individual-subject level, (1) higher-order regression models [e.g., Friston 24-parameter model (Friston et al., 1996)] perform better than lower-order models, (2) including scrubbing approaches is superior to regression models alone, and (3) global signal regression controls for head motion more than any approach attempting to explicitly model motion. Importantly, several studies have suggested that despite the best of efforts, motion cannot be fully accounted for at the individual-subject level and argued that motion may be better accounted for at the group-level (i.e., covariate analysis) when possible (Fair et al., 2012; Satterthwaite et al., 2013; Yan et al., 2013a). While some, or all, of these findings may generalize to graph theoretical analyses, this remains an open issue.

Here, we extend our prior work that examines the impact of motion on seed-based correlation analyses and regional R-fMRI measures (Yan et al., 2013a) to include topological properties. Consistent with our prior work, we assess not only the impact of motion on topologic measures and findings of inter-individual and group-related differences, but the ability of previously established motion correction procedures to account for the confounding effects of motion (see Table 2). Importantly, when considering graph theoretical analyses, it is essential to appreciate the potential impact of motion on graph construction, prior to the derivation of topologic measures. In order to address this concern, we begin our examination with an analysis of the impact of motion on the density of graphs derived through correlation coefficient thresholding. Additionally, for all topologic measures and procedures examined, we systematically vary density to establish the robustness of our findings.

**Table 2:** **Head motion correction strategies investigated in the current study**.

**Correction strategies**	**Descriptions**
**INDIVIDUAL-LEVEL**	
***Preprocessing-stage:* motion was corrected during preprocessing**	
Rigid-body 6	Regress out 6 head motion parameters
Friston 24	Regress out 6 head motion parameters, 6 head motion parameters one time point before, and the 12 corresponding squared items (Friston et al., 1996)
Friston 24 + scrubbing	Identifying “bad” time points using a threshold of FD (Power) >0.2 mm as well as 1 back and 2 forward neighbors as done in Power et al. (2013), then modeling each “bad” time point as a separate regressor in the regression models
Polynomial regression	The difference in correlation values (Δ*r*) were calculated between the *r*-values acquired in Strategy 2 (Friston 24) and those in Strategy 3 (Friston 24 + scrubbing). A 5-degree polynomial model was used to fit Δ*r* values on Euclidean distances, and then these fitted Δ*r* values were regressed out from the original *r* values (Fair et al., 2012)
***Connection estimation-stage:* motion was corrected during connection estimation**	
Graphical lasso	Using graphical lasso to estimate the partial correlation instead of full correlation
**GROUP-LEVEL**	
*Connection-stage:* the following motion-related parameters were regressed out from each connection before topological parameter calculation	
Mean iFC regression	Whole brain mean iFC
Motion regression	Mean FD
Mean iFC + motion regression	Both mean iFC and mean FD
***Topological parameter-stage:* the following motion-related parameters were regressed out from each topological parameter after their calculation**	
Mean iFC regression	Whole brain mean iFC
Motion regression	Mean FD
Mean iFC + motion regression	Both mean iFC and mean FD
***Both stages:* the following motion-related parameters were regressed out from each connection before topological parameter calculation, as well as from each topological parameter after their calculation**	
Mean iFC regressed	Whole brain mean iFC
Motion regressed	Mean FD
Mean iFC + motion regressed	Both mean iFC and mean FD

FD, framewise displacement; iFC, intrinsic functional connectivity.

## Methods

### Participants and imaging protocols

We performed our analyses on publicly available imaging data from the 1000 Functional Connectomes Project (FCP; data are available at http://fcon_1000.projects.nitrc.org). Consistent with our previous study (Yan et al., 2013a), data of 176 participants (70 males, 20.9 ± 1.9 years) in the Cambridge dataset were used in our main analyses. In addition, data of 176 participants (70 males, 21.2 ± 1.9 years) in the Beijing dataset were used to assess the generalizability of our main analyses. The corresponding institutional review boards approved or provided waivers for the inclusion of anonymized data in the FCP. Data were acquired with written informed consent from each participant.

Participants were instructed to simply rest while awake in a 3T scanner, and R-fMRI data were acquired using an echo-planar imaging (EPI) sequence (Cambridge dataset: repeat time (TR) = 3 s, echo time (TE) = 30 ms, time points = 119, slice number = 47, voxel size = 3 × 3 × 3 mm^3^, field of view (FOV) = 216 × 216; Beijing dataset: *TR* = 2 s, *TE* = 30 ms, time points = 235, slice number = 33, voxel size = 3.12 × 3.12 × 3.6 mm^3^, FOV = 200 × 200). A high-resolution T1-weighted magnetization prepared gradient echo image (MPRAGE) was also obtained for each participant to perform spatial normalization and localization.

### Preprocessing

Unless otherwise stated, all preprocessing was performed using the Data Processing Assistant for Resting-State fMRI (DPARSF, Yan and Zang, 2010, http://www.restfmri.net), which is based on Statistical Parametric Mapping (SPM8) (http://www.fil.ion.ucl.ac.uk/spm) and Resting-State fMRI Data Analysis Toolkit (REST, Song et al., 2011; http://www.restfmri.net), running in Matlab R2012a (Natick, MA). All volume slices were corrected for different signal acquisition times by shifting the signal measured in each slice relative to the acquisition of the slice at the mid-point of each TR. Then, the time series of images for each subject were realigned using a six-parameter (rigid body) linear transformation with a two-pass procedure (registered to the first image and then registered to the mean of the images after the first realignment). Individual structural images (T1-weighted MPRAGE) were co-registered to the mean functional image after realignment using a 6 degrees-of-freedom linear transformation without re-sampling. The transformed structural images were then segmented into gray matter (GM), white matter (WM) and cerebrospinal fluid (CSF) (Ashburner and Friston, 2005). The Diffeomorphic Anatomical Registration Through Exponentiated Lie algebra (DARTEL) tool (Ashburner, 2007) was used to compute transformations from individual native space to MNI space.

### Head motion correction strategies (individual-level)

As identified in our previous study (Yan et al., 2013a), the Friston 24-parameter model performed well in addressing head motion effects, which is consistent with other studies that found higher-order models performed better than lower-order models (Satterthwaite et al., 2013; Power et al., 2014). Thus, we compared the following individual-level correction strategies at the preprocessing-stage in the current study (see Table 2):

1. Regression of realigned data on 6 head motion parameters (i.e., three translations and three rotations) (rigid-body 6-parameter model);
2. Regression of realigned data on 6 head motion parameters, 6 head motion parameters from the previous time point, and the 12 corresponding squared items (Friston et al., 1996) (Friston 24-parameter model);
3. Scrubbing within Friston 24-parameter model regression (spike regression): “bad” time points were identified using a threshold of framewise displacement (FD, Power et al., 2012) > 0.2 mm as well as 1 back and 2 forward neighbors as performed by Power et al. (2013), then each “bad” time point was modeled as a separate regressor in the regression models (Lemieux et al., 2007; Satterthwaite et al., 2013; Yan et al., 2013a) in addition to Strategy 2 (Friston 24 + scrubbing);
4. Recently, Fair et al. (2012) proposed a method that incorporates the information of scrubbing but does not result in a reduction of degrees of freedom. The difference in correlation values (Δ*r*) were calculated between the correlation (*r*)-values acquired in Strategy 2 and those in Strategy 3. A 5-degree polynomial model was used to fit Δ*r* values on Euclidean distances, and then these fitted Δ*r* values were regressed out from the original *r*-values acquired in Strategy 2 (Polynomial regression).

As scrubbing can result in the removal of a large number of time points (Power et al., 2012, 2013; Satterthwaite et al., 2013; Yan et al., 2013a), to obtain reliable results, we removed subjects who had less than 3 min of data remaining after scrubbing, as done in our previous study (Yan et al., 2013a). This resulted in the exclusion of 18 subjects in the Cambridge datasets from the main analyses, leaving 158 subjects for these analyses.

### Global signal regression (GSR)

GSR is a commonly used, yet controversial practice in the R-fMRI field, that yields substantial increases in negative correlations (Murphy et al., 2009; Weissenbacher et al., 2009) and may distort group differences in intrinsic functional connectivity (iFC) (Saad et al., 2012, 2013; Gotts et al., 2013). However, recent studies have found that GSR is more effective in removing relationships between motion and correlation-based R-fMRI metrics across subjects than any correction strategy that explicitly models motion (Yan et al., 2013a; Power et al., 2014). Thus, we evaluated the effects of head motion correction strategies on analyses performed with and without GSR.

Within the nuisance regression step, linear and quadratic trends were included as regressors to account for low-frequency drifts, and signals from WM and CSF were regressed out to reduce respiratory and cardiac effects, in the BOLD signal.

After nuisance regression, the functional data were transformed to MNI space and resampled to 3 × 3 × 3 mm^3^ voxel size with DARTEL tool (Ashburner, 2007). Spatial smoothing was not performed to avoid mixing signals between different regions (see section Network Construction). Temporal filtering (0.01–0.1 Hz) was then applied to the time series of each voxel to reduce the effect of low-frequency drifts and high-frequency noise.

### Network construction

The connectome graph is composed of distinct brain regions (nodes) and their functional interactions (edges). The whole brain was first parcellated into 90 cortical and subcortical regions of interest (45 for each hemisphere, see Table A1) using a prior anatomical automatic labeling (AAL) atlas (Tzourio-Mazoyer et al., 2002). Although the AAL atlas is widely used in brain network topology analysis, Smith et al. (2011) demonstrated the use of functionally inaccurate ROIs is damaging to network estimation, and thus suggests against structural atlases. Here we also evaluated the networks based on two functional atlas for supplementary analyses: Dosenbach's 160 ROIs which were generated based on meta-analysis (Dosenbach et al., 2010), and Craddock's 200 ROIs which were generated based on spatially constrained spectral clustering (Craddock et al., 2012).

The mean time series of each region was extracted by averaging the time series of all voxels within that region. Pearson's correlation coefficients were estimated for each pair of regions and were transformed to Fisher's *z*-score (Fisher, 1915) to create the iFC matrix for each participant. The correlation matrices were further thresholded into binary networks or weighted networks to examine the head motion impact on binarized topology or weighted topology. Two thresholding strategies are widely used: correlation coefficient thresholding and density thresholding; each has its own limitations (Fornito et al., 2013). The correlation coefficient thresholding strategy resulted in networks with densities (the number of existing edges divided by the maximum possible number of edges) that are sensitive to head motion (see results in the section “Head Motion Impact on Graph Construction”); this in turn affects the topological properties. As such, we used the density thresholding strategy to normalize the number of edges among all of the graphs. A wide range of density thresholds (2% ≤ density ≤ 50%, step of 2%) was chosen to allow prominent small-world properties in brain networks to be observed (Watts and Strogatz, 1998) (for details, see the Results section).

While the primary focus of the present work is on graphs derived using full correlation (Pearson's correlation), we also felt that it is important to address potential differences when partial correlation-based graphs are used instead. Partial correlation-based approaches should inherently remove signals present throughout the brain; as such, we predicted that graphs generated from partial correlation should be more robust to motion. Of note, a key limitation for partial correlation approaches is that the covariance matrix is not invertible for most R-fMRI datasets due to the limited number of time points relative to the large number of nodes. This challenge is compounded by additional losses in the number of degrees of freedom produced by temporal filtering. In order to address this, we utilized the graphical lasso method to estimate the sparse inverse matrix through L1 norm (lasso) regularization (Friedman et al., 2008) (http://www-stat.stanford.edu/~tibs/glasso/). We systematically varied the regularization penalties^1^ to acquire matrices with the desired density (2% ≤ density ≤ 50%, step of 2%) for each participant.

### Network analysis

We investigated both the global and regional topological properties of brain graphs (Table 1). At the global level, we investigated local efficiency, global efficiency, clustering coefficient, characteristic path length, normalized clustering coefficient, normalized characteristic path length, small-worldness, assortativity and modularity. At the regional level, we computed degree centrality, nodal efficiency, nodal clustering coefficient, subgraph centrality, betweeness centrality and eigenvector centrality for each node.

All of the topological parameters investigated in the current study are summarized in Table 1, and were calculated with the Brain Connectivity Toolbox (Rubinov and Sporns, 2010) (http://www.brain-connectivity-toolbox.net). For details about the computation of network parameters, please see (Rubinov and Sporns, 2010).

### Statistical analysis

To examine head motion effects on the topological properties of the connectome graph, we calculated the correlation between head motion and each of the parameters across participants. Head motion was indexed by mean FD derived with Jenkinson's relative root mean square (RMS) algorithm (Jenkinson et al., 2002); mean FD (Jenkinson) was used due to its consideration of voxel-wise differences in motion in its derivation (Yan et al., 2013a).

To investigate the need for group-level motion correction after individual-level correction (Fair et al., 2012; Satterthwaite et al., 2012; Van Dijk et al., 2012; Yan et al., 2013a), we also compared topological parameters derived from subjects in the upper and lower terciles of head motion, as in our prior study (Yan et al., 2013a). The upper and lower motion terciles were created using only females (*n* = 32 / group) to avoid potential confounds associated with sex; age did not differ. Two-sample *t*-tests were performed between the two motion groups to test motion effects with and without group-level correction.

Group-level corrections were performed at two stages: connection-stage and/or topological parameter-stage (Table 2). For each stage, two kinds of regressors were regressed out: mean iFC and/or mean FD. The regression of mean iFC is motivated by its ability to address unwanted additive noise as demonstrated in our prior work on standardizing R-fMRI measures (Yan et al., 2013b).

## Results

### Head motion impact on graph construction

**Figure 1:**
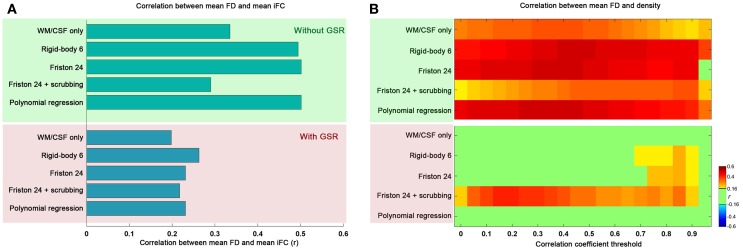
**The relationship between mean framewise displacement (FD) and mean intrinsic functional connectivity (iFC) (A) as well as density under different correlation thresholds (B).** The mean iFC was averaged across all the connections between all the pairs of 90 ROIs of automated anatomical labeling (AAL) atlas. Five preprocessing strategies were evaluated in combination without global signal regression (GSR: green shaded) and with GSR (pink shaded). The correlation is considered significant at *p* < 0.05 (|*r*|> 0.16). We did not correct for multiple comparisons to avoid false negative effects of head motion.

Topological parameters derived from graph theoretical analyses are highly sensitive to graph construction. In order to address concerns regarding the potential impact of motion on graph construction, we examined the relationship between mean FD and mean iFC (calculated by averaging the Fisher's *z* value across all connections for an individual). Our findings indicate that mean iFC is highly correlated with motion when GSR is excluded, regardless of the motion correction strategy employed; in contrast, when GSR is applied, mean iFC relationships with motion were more moderate (Figure 1A).

We also examined the impact of motion on the density of graphs derived using the correlation coefficient thresholding strategy. As would be expected, the global increase in iFC with motion results in increased density, regardless of the r threshold applied for graph construction (Figure 1B). Once again, we found GSR to be a major determinant of our findings, with graph density exhibiting markedly greater relationships (across correlation thresholds) with motion when the data were processed without GSR, rather than with GSR (which diminished nearly all relationships between graph density and motion, regardless of motion correction approaches employed). This is consistent with our prior finding that GSR controls for head motion more than any approach attempting to explicitly model motion (Yan et al., 2013a).

One other consideration that should be noted is the impact of scrubbing on motion-density relationships. Specifically, we found that scrubbing reduces motion-density relationships the most among the individual-level correction strategies when GSR is not used. This benefit was not seen when GSR is used—in fact, the combination of scrubbing and GSR appeared to increase motion relationships relative to GSR alone. This may at first appear to be surprising, but it is important to note that participant data requiring a higher degree of scrubbing will inherently have a higher likelihood of extreme correlation values after scrubbing due to decreases in the number of degrees of freedom; this in turn will increase density (i.e., more edges) (Yan et al., 2013a)—please see an expanded discussion in the section “Reviving or Learning from Global Signal Regression?”

Overall, these results indicate that one should be extremely cautious when using a correlation- or *p*-value-based threshold to construct brain graphs, as the results can be highly confounded by head motion; GSR can alleviate these concerns. Nonetheless, given the impact of head motion on graph construction with correlation-based thresholding, our remaining analyses were carried out using a density thresholding strategy in which the number of graph connections across participants and processing strategies was normalized. We report our main results based on binarized graphs, though our analyses using weighted graphs yielded similar results (see section “Generalizability of Findings”).

### Motion-robust small-world properties in the connectome graph

**Figure 2:**
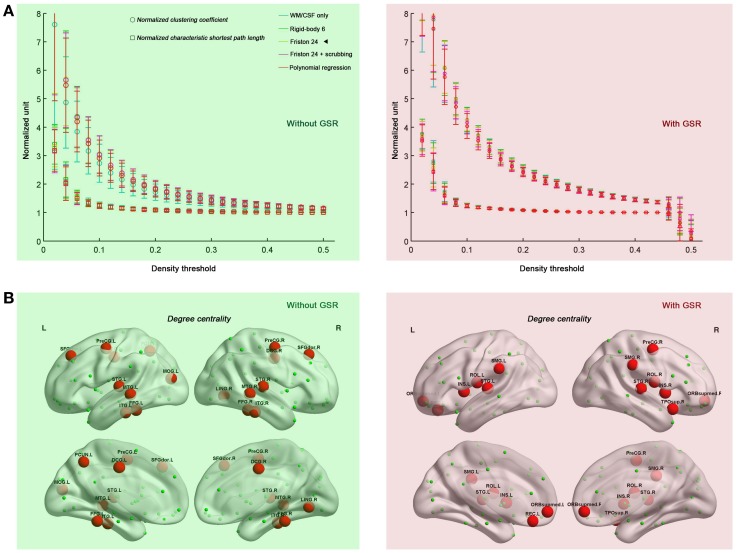
**Small-world properties (A) and hub distributions (B) under different head motion correction strategies.** Five preprocessing strategies were evaluated in combination without GSR (green shaded) and with GSR (pink shaded). “◄” in panel **(A)** indicates that the hub distribution demonstrated in panel **(B)** is derived from Friston 24 model. The hub distribution was demonstrated with the area under the curve (AUC) of degree centrality integrated within density range of 0.06–0.44, while regions with AUC > mean + SD are considered as hubs. For the abbreviations of the regions, see Table A1. Surface maps were created with BrainNet Viewer (Xia et al., 2013 www.nitrc.org/projects/bnv/).

Prior work has demonstrated that human connectome graphs based upon iFC follow a small-world topology (i.e., high clustering and short path lengths linking different nodes) (Salvador et al., 2005; Achard et al., 2006; Achard and Bullmore, 2007; Liao et al., 2011; Yan and He, 2011; Yu et al., 2011). Here, we tested whether the prominent small-world architecture is robust to the various head motion correction strategies, finding that the graphs derived from all the correction strategies retained small-world properties, independent of density level (0.06–0.44) (Figure 2A). When compared with 100 random networks with the same number of nodes, edges, and degree distribution as the observed graph (Maslov and Sneppen, 2002), the brain networks had an almost identical path length (normalized characteristic shortest path length ~1) but were more locally clustered (normalized clustering coefficient >1). Taken together, the current results indicate the previous findings of small-world properties in human functional networks cannot be easily attributed to the presence of head motion. As will be discussed in the following sections, this statement is not intended to imply that head motion does not impact topological parameters.

**Figure 3:**
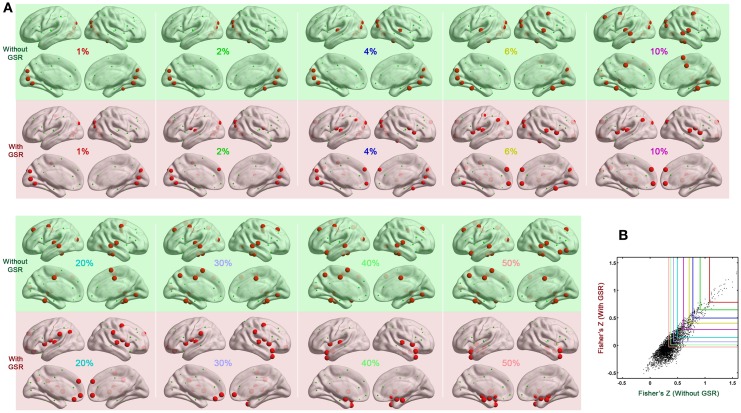
**Impact of density on hub distribution. (A)** Hub distribution across various densities either without GSR (green shaded) or with GSR (pink shaded) derived from the data corrected with Friston 24 model. With stringent density thresholds, the hub distributions are similar between data with and without GSR. When the density increases, the discrepancy of hub distribution between with and without GSR becomes dominant. **(B)** Scatter plot of Fisher's Z averaged across participants. Most of the top connections can be identified either with or without GSR. However, when the percentage increases, a large portion of connections can be only identified by one procedure but not the other.

We also tested if hub distribution is robust to head motion correction strategies. We first calculated node degree centrality over the range of densities that maintained small-worldness, i.e., 0.06–0.44, and then calculated the area under curve (AUC) for this range. The AUC of degree centrality was averaged across all the participants, and regions with degree > mean + one standard deviation (SD) across nodes were identified as hubs (Figure 2B). Head motion correction strategies had little impact on the identification of hubs, though once again, the presence of absence of GSR was a major determinant of findings. In the case without GSR, the hubs were predominantly attributed to fronto-parietal network and temporal regions, while shifted into default mode network and insula in the case with GSR. However, there is an important caveat on this finding if one looks at motion-hub distribution relationships for individual density levels, rather than using AUC. The hub distributions are similar between data with and without GSR when the density is low (<6%); however, when the density increases, the discrepancy of hub distribution between with and without GSR becomes dominant (Figure 3A). This can be explained by the alteration in correlation distribution induced by GSR (Figure 3B). The top percentage of connections can be identified either with or without GSR. However, the weaker connections identified will differ as a function of whether or not GSR is applied. In sum, GSR is not only mean-centering the intrinsic connectivities, but can also affect their relative structure as well as hub distribution.

### Head motion impact on global topological properties

**Figure 4:**
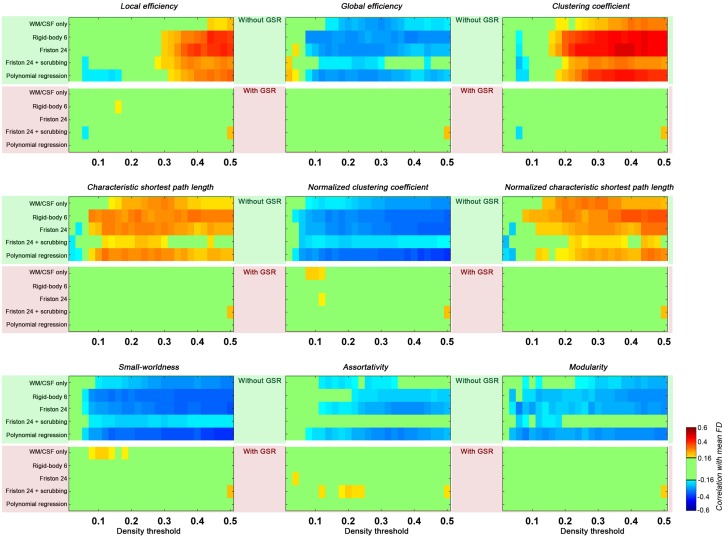
**Correlations between head motion and global topological properties.** Each panel presents one property. The upper green shaded section is without GSR and the lower pink shaded section is with GSR. Each row represents a preprocessing strategies and each column represents a density threshold. The correlation is considered significant at *p* < 0.05 (|*r*|> 0.16). We did not correct for multiple comparisons to avoid false negative effects of head motion.

Head motion increased local efficiency while decreasing global efficiency (Figure 4). These findings generalized across nearly all densities above 0.1 for global efficiency, but were limited to densities greater than 0.3 for local efficiency. Of note, here the topological properties were derived from graph constructed with density threshold; in other words, relationships with head motion exist in network structure even when the wiring cost (i.e., number of connections) is controlled. When GSR is performed, such head motion relationships are removed.

With regard to small-worldness, we found that motion is negatively associated with small world properties—a finding that generalized across density levels greater than 0.1, and was once again diminished with GSR. To interpret these findings, it is important to understand the impact of motion on the two constituent measures for small-worldness—the normalized clustering coefficient and the normalized characteristic shortest path length. As previously described, higher head motion is associated with an increase in local efficiency (which is equivalent to clustering coefficient) of the constructed graph, and also for degree-matched random networks. The increase in clustering coefficient of the constructed network is less than the increase in degree-matched random networks, leading to a negative correlation between head motion and normalized clustering coefficient. In contrast, the characteristic shortest path length (the inverse of global efficiency) and its normalized version (compared to random networks) were both positively correlated with head motion. Combining the normalized clustering coefficient and normalized characteristic path length, the small-worldness was negatively correlated with head motion. Once again, such an effect is significant in the case without GSR, but almost completely diminished by GSR.

### Head motion impact on regional topological properties

**Figure 5:**
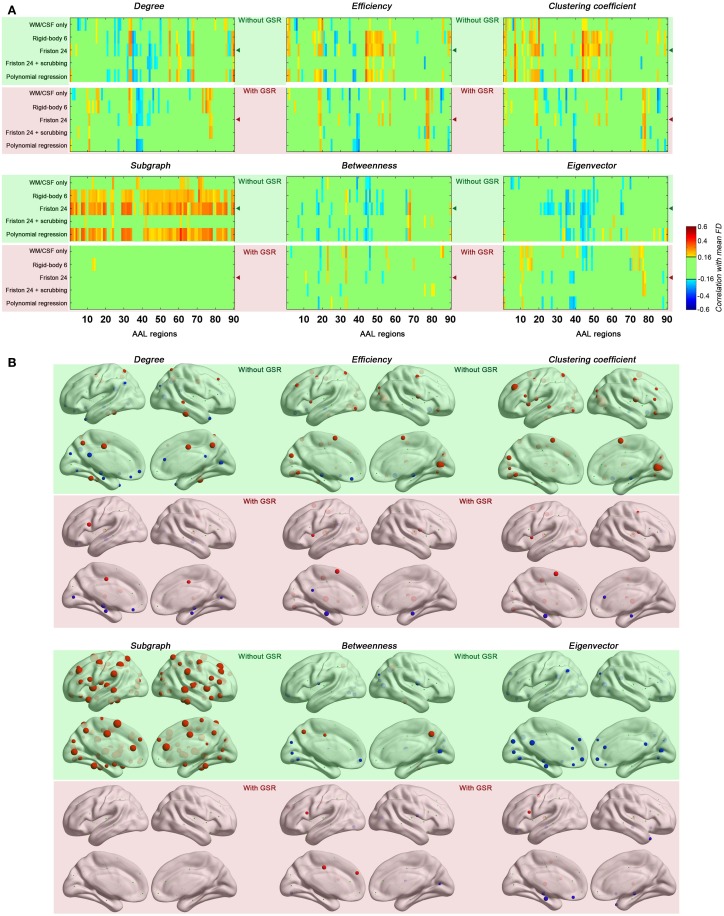
**Correlations between head motion and regional topological properties were plotted in matrix (A) and on brain surface (B).** The layout of panel **(A)** is the same as Figure 4 except that each column represents one of the AAL regions. The regional properties were characterized by the area under the curve (AUC) of each measure integrated within density range of 0.06–0.44 and the head motion correlation with these AUCs was demonstrated in panel **(B)**. The size of spheres denotes the strength of correlation, red spheres denote positive correlations, blue spheres denote negative correlations, and green spheres denote insignificant correlations (*p* > 0.05, |*r*|< 0.16). “◄” in panel **(A)** indicates that the node correlation demonstrated in panel **(B)** is derived from Friston 24 model.

**Figure 6:**
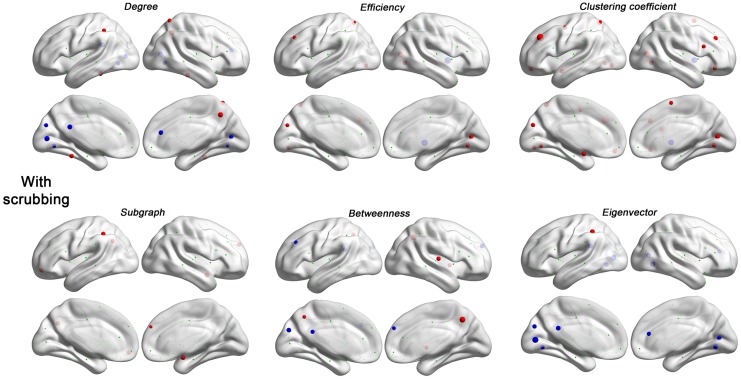
**Head motion impact on six regional topological properties with scrubbing (Friston 24 + scrubbing) at FD (Power) > 0.2 mm.** The regional properties were characterized by the area under the curve (AUC) of each measure integrated within density range of 0.06–0.44. The head motion correlation with these AUCs was demonstrated. The size of spheres denotes the strength of correlation, red spheres denote positive correlations, blue spheres denote negative correlations, and green spheres denote insignificant correlations (*p* > 0.05, |*r*|< 0.16).

Next, we evaluated the impact of head motion on regional topological properties; the AUC densities in the range of 0.06–0.44 were used as in section Motion-Robust Small-World Properties in the Connectome Graph. In our prior work, we found degree centrality was drastically increased with motion, and that relationships with motion were markedly reduced by GSR or Z-standardization (i.e., mean centering + variance normalization) (Yan et al., 2013a). Unlike our previous findings, which were based on a *p*-value-based thresholding strategy (similar to correlation coefficient thresholding), here we found that with density thresholding (i.e., the mean degree was controlled accordingly), both positive and negative relationships with motion were noted for region-wise degree centrality, depending on the specific region examined (Figure 5). Interestingly, the degree centralities of precuneus, precentral, fusiform, middle temporal, median cingulate and paracingulate gyri—the hub regions when no GSR is used—were positively correlated with head motion. On the other hand, the degree centralities of default mode network regions—medial prefrontal cortex (MPFC), posterior cingulate cortex (PCC), angular gyrus, hippocampus and parahippocampal gyrus—were negatively correlated with head motion. Such findings are in line with our prior findings that head motion is positively associated with motor cortex and negatively correlated with the default mode network (Yan et al., 2013a). Of note, head motion associations decreased with scrubbing, but the pattern was similar (i.e., no new regional associations emerged) (Figure 6). A key challenge in the interpretation of these findings, which was discussed previously and will be expanded in our discussion, is determining whether or not the motion–BOLD relationships are purely artifactual, or may in part reflect motion-related neural activity or indices of kinetic traits.

Regarding regional topological properties, which reflect local properties, e.g., nodal efficiency and nodal clustering coefficient, we generally found positive relationships with head motion. However, the pattern was reversed for the topological properties that reflect global properties, e.g., betweenness and eigenvector centrality. Subgraph centrality, a measure considered to reflect middle- or meso-scale properties (Zuo et al., 2012), was drastically increased with motion. These findings are consistent with our findings that head motion increased local efficiency while decreased global efficiency (see prior section). Once again, when time points with relatively larger frame-wise displacements were removed via scrubbing, relationships with head motion observed for the various centrality measures were reduced, though the overall patterns remained (Figure 6).

When GSR was included in preprocessing, relationships between head motion and regional topological properties were diminished. It is important to note that since we controlled density in our graph construction step, the same amount of highly connected edges were present in the cases of processing with and without GSR—thus removing a major potential confound. The markedly different motion relationships noted with GSR suggest that GSR is not just mean-centering correlation scores, but also altering the connectivity structure. The manner in which GSR alters this structure remains largely unknown.

### The impact of graphical lasso on head motion relationships

**Figure 7:**
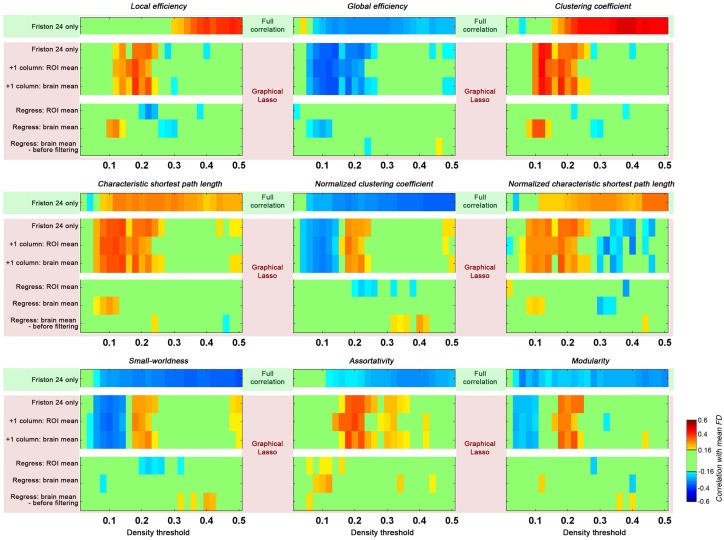
**Impact of graphical lasso (partial correlation) on head motion relationships with global topological parameters.** The head motion effects of full correlation was demonstrated as a base (green shaded, based on Friston 24 model), while different implementation of graphical lasso was implemented based on the Friston 24 model (pink shaded). Two scenarios were created to test the GSR effect with graphical lasso: (1) the global signal was added as an additional time series to the parcellation set (+1 column); (2) the global signal was regressed out of the fMRI time series data prior to performing graphical lasso (Regress). The GS time series was calculated by averaging the time series across either all ROIs (ROI mean), or all voxels (brain mean). For the second scenario, the brain mean was regressed out in cases of either before or after filtering.

When partial correlation (using graphical lasso) was utilized instead of full correlation for estimating connections, we found that topological parameters were insensitive to motion effects at higher density thresholds (e.g., >0.25) as compared to those based on full correlation (Figure 7). However, head motion effects were more prominent for lower densities (0.05–0.25) when graphical lasso was employed. These results indicate that although graphical lasso removes the variance of other regions when estimating the relationship between two specific regions, it did not remove the “global effect” as addressed by GSR.

Given that we found GSR diminished the relationship between head motion and global topological properties, we tested the effect of GSR on graphical lasso estimates of connectivity using two strategies: (1) the global signal was added as an additional timeseries to the parcellation set; (2) the global signal was regressed out of the fMRI timeseries data prior to performing graphical lasso. In the first case, when the GS timeseries was treated as a signal akin to any ROI's timeseries, the result was identical to those obtained from graphical lasso without the GS timeseries. Of note, this finding did not depend on whether the GS timeseries was calculated by averaging the timeseries across all ROIs, or all voxels. In contrast, regressing out the GS prior to carrying out graphical lasso reduced the effect of head motion as previously seen with full correlation. Once again, we found that this did not depend on the specific approach used to calculate the GS; additionally, it did not matter if the GS was regressed before or after filtering. Given that the GS should be theoretically removed by partial correlation or graphical lasso itself, it is not clear why GSR prior to graphical lasso has such an impact.

### Group level correction in addressing residual head motion impact

Previous studies have suggested the necessity of accounting for motion at the group-level when possible (Fair et al., 2012; Van Dijk et al., 2012; Satterthwaite et al., 2013; Yan et al., 2013a). While these reports primarily highlighted the merits of including mean FD as a covariate in group-level analyses, more recent work has suggested additional benefits of correcting each participant's data for global distribution parameters (e.g., the mean R-fMRI for each individual) (Saad et al., 2013; Yan et al., 2013b). Here, we explored the group-level correction targeting two different stages: (1) *the connection*—for each edge, we regressed the correlation scores across subjects on their mean iFC scores and/or motion, and then perform graphical theoretical analysis, (2) *topological parameter*—we added mean iFC and/or mean FD as covariates in group analysis after the topological parameters are calculated. Following the approach of our prior work (Yan et al., 2013a), this was accomplished by comparing a “high”-motion vs. a “low”-motion participant group; the upper and lower-motion terciles of females in the publically available Cambridge dataset were used to define these two groups.

**Figure 8:**
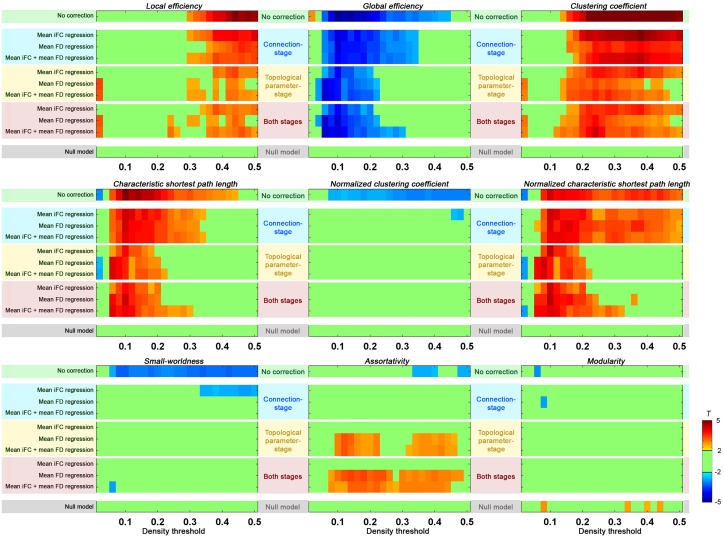
**Group-level correction in addressing residual head motion impact.** Two-sample *t*-tests were performed on each of the global topological properties between upper motion tercile (*n* = 32) and lower motion tercile (*n* = 32) of Cambridge females. The “null model” was defined by performing two-sample *t*-test between two “null” groups (mixing the motion terciles and equating for motion and age). The group-level correction was performed either at connection-stage (i.e., applied to the connections before topological parameter calculation), at topological parameter-stage (i.e., applied to the topological parameters after their calculation), or at both stages. For each stage, the mean iFC, mean FD, or both mean iFC + mean FD were regressed out.

In order to carry out group-level correction on global distribution parameters, we first needed to calculate mean iFC. While these values can be calculated from the mean iFC across all ROIs for each participant, as done in the section “Head Motion Impact on Graph Construction”, the results can be biased by the atlas used. Here we estimated the mean iFC between all the voxel-to-voxel connectivities across the brain (70831 voxels) to avoid such a bias^2^; as expected, the measure was highly correlated with head motion across subjects (*r* = 0.51, *p* < 10^−11^). The following connection-stage corrections were performed and compared: (1) mean iFC regressed; (2) motion (mean FD) regressed; (3) (mean iFC + mean FD) regressed. Consistent with the goal of removing unintended, but systematic, global variations across subjects, mean iFC regression reduced the motion effect when compared to non-correction (Figure 8). Directly regressing out head motion from the edges across subjects produced even greater reductions in motion effects. When we regressed out both mean iFC and mean FD the head motion effects were reduced in a similar extent, but this may have the additional benefit of addressing unwanted global variations beyond head motion.

When we performed the group-level correction of topological parameters by including mean iFC and/or mean FD as covariates (topological parameter-stage), significant reductions were noted in the difference between the high motion and low motion terciles. This reduction was significant as compared to uncorrected data, and even compared to the connection-stage group-level correction. We further combined group-level correction at both stages, but without clear benefit as compared to the topological parameter-stage correction.

### Generalizability of findings

**Figure 9:**
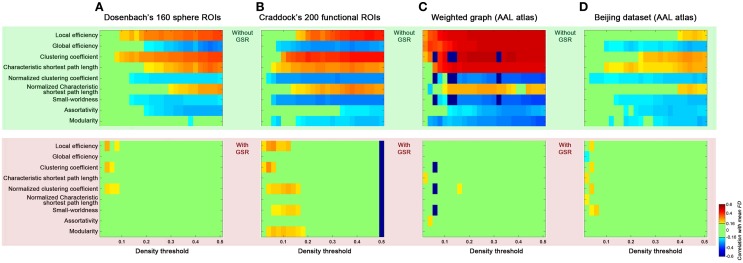
**Generalizability of the current findings of head motion dependencies.** We addressed possible concerns regarding the generalizability of our findings to other studies by computing the topological parameters based on parcellation set (**A**: Dosenbach's 160 sphere ROIs, **B**: Craddock's 200 ROIs), weighted graph based on AAL atlas **(C)**, and Beijing dataset based on AAL atlas **(D)**. Results without GSR were shaded in green and with GSR were shaded in pink.

Finally, we addressed possible concerns regarding the generalizability of our findings to other studies by varying several factors (Figure 9): (1) brain parcellation approach; (2) connection type (binary vs. weighted); (3) dataset (Cambridge vs. Beijing). First, we examined the effect of parcellation approach on our findings by repeating our analyses with brain graphs constructed from Dosenbach's 160 spherical ROIs that were generated based on a meta-analysis (Dosenbach et al., 2010) (Figure 9A), and Craddock's 200 ROIs that were generated based on spatially constrained spectral clustering (Craddock et al., 2012) (Figure 9B). Similar to our findings with AAL, for these two parcellations, we found head motion effects on the global topological parameters in the case without GSR; such relationships were diminished when GSR was employed. Next, we examined the impact of connection type, by repeating our analyses using weighted connections, finding the effect of head motion on the global topological parameters were once again significant without GSR, and diminished when GSR was employed (Figure 9C). Finally, we repeated our analyses using the Beijing dataset; the findings generalized well from the Cambridge dataset, further increasing our confidence in them (Figure 9D).

## Discussion

The present work provides a comprehensive examination of the relationship between inter-individual differences in commonly used topological parameters and motion, yielding multiple important findings. First, we found that head motion increases iFC throughout the brain, and as such, confounds graph construction when correlation (*p*-value) based thresholds are employed to determine the presence of edges. Density thresholding was used as a means of avoiding this potential confound in the present work. As expected, small-world properties were related to the presence of head-motion, though could not be attributed to motion alone (i.e., small world properties persist after motion correction). Consistent with our prior work, global signal regression proved beneficial with respect to its ability to mitigate relationships between topological properties of the connectome graph and head motion. Consistent with its ability to remove globally present signals, using partial correlation to estimate graph connections also reduced the influences of motion on topological parameters, although not to the degree observed with GSR. Finally, it is worth noting that, consistent with our prior work, group-level corrections were effective in reducing motion relationships for topologic parameters, although they were more effective when applied after graph topological parameter calculation (i.e., as covariates in group level analyses for topological parameters). Importantly, we found that our findings generalized across parcellation sets, connection types (binary, weighted) and datasets.

### Motion-dependencies in graph construction

Motion poses a distinct challenge for graph theoretical R-fMRI measures, as it confounds construction of the graph upon which the parameters are based by inflating the number of edges. The increased wiring cost associated with motion in turn biases topological parameters, regardless of whether they are global or regional. Central to any effort to minimize the relationship between topological parameters and motion, is the minimization of its impact on graph construction. In this regard, we found density thresholding to be superior to correlation or *p*-value thresholding as it fixes the number of connections in the brain across participants. This avoids motion-related variation in the number of connections from one participant to the next, which are present when correlation thresholding strategies are employed due to increases in correlation levels throughout the brain inherently produced by motion. However, density thresholding has its own limitations. First, it results in a loss to the biological validity of the analysis, as it is highly unlikely that all individuals have the same number of connections in their brain. Second, the specific correlation threshold making the top n% connections varies across subjects (Fornito et al., 2013) and can be affected by motion and preprocessing strategy decisions—particularly when higher density threshold are employed.

The present work draws attention to group-level correction as a means of accounting for the influences of motion on graph construction and topological parameters. Such approaches can be applied to individual connections prior to graph construction, or to topological parameters calculated after graph construction. Regressing mean iFC and mean FD from each connection prior to graph construction can effectively remove motion-density relationships with respect to correlation and *p*-value thresholding (the correlation between mean FD and density across *r* thresholds are within −0.02 to 0.05), while allowing the density to vary across participants. Although potentially less obvious, our analyses suggest that graph construction with density thresholding is affected by motion as well, and can benefit group-level correction of individual connections prior to graph construction. An interesting finding of the present work is that connection-level corrections cannot entirely remove motion dependencies for topologic parameters, necessitating group-level covariate analysis for topologic parameters.

### Reviving or learning from global signal regression?

Consistent with prior work (Yan et al., 2013a), the most robust finding of the present work was the ability of GSR to remove motion-relationships for R-fMRI metrics. This may at first seem to be a vindication of GSR, or at least an argument for resurgence of usage of GSR, which has decreased in the small world literature in recent years without replacement by an alternative technique for handling motion.

**Figure 10:**
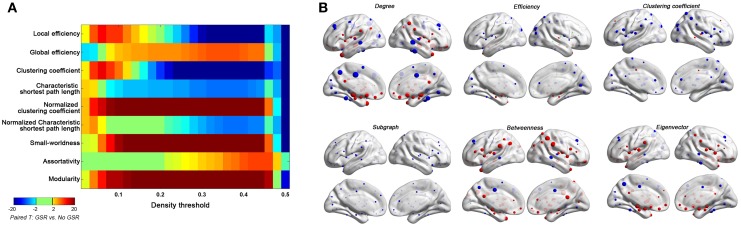
**Impact of GSR on global topological properties (A) and regional topological properties (B) based on Friston-24 model.** In panel **(B)**, the regional properties were characterized by the area under the curve (AUC) of each measure integrated within density range of 0.06–0.44. The GSR effect was evaluated by paired *t*-test on these AUCs. The size of spheres denotes the strength of difference, red spheres denote GSR increased the property, blue spheres denote GSR decreased the property, and green spheres denote insignificant effect (*p* > 0.05, |T|< 2).

Unfortunately, the picture for GSR is not that simple. Prior demonstrations of the potential for GSR to artifactually exaggerate or introduce negative correlation coefficients (Murphy et al., 2009; Weissenbacher et al., 2009), as well as artifactually alter the covariate structure in group-level analyses (Saad et al., 2012, 2013; Gotts et al., 2013), cannot go unheeded. Nor can concerns about potential difficulties in interpretation of findings with GSR as it's actually GM signal regression (Yan et al., 2013b). In the present work, we found that GSR can do more than just mean-centering data, as the specific connections surviving density thresholding can change with the presence of GSR (regardless of whether aggressive motion corrections, such as scrubbing, were applied)—in turn producing drastic changes in topological parameters as well as hub distributions (Figure 10). Our findings suggest that one way to obtain topological properties and hub distributions that are robust to preprocessing strategy, is to adopt a more stringent density threshold (e.g., <6%) at which only the top connections survive—as these are the same with or without GSR. One notable caveat in this suggestion is that given our lack of knowledge concerning the true wiring cost of the brain, stringent thresholding may or may not compromise biological validity and/or sensitivity.

**Figure 11:**
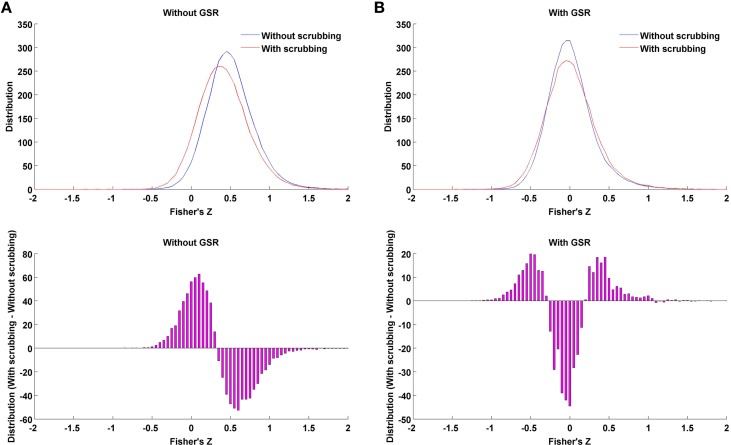
**Impact of scrubbing on correlation distribution.** Twenty-five subjects with highest motion (to allow a number of time points being scrubbed) of the 158 subjects were selected to form the mean distribution in the case of without GSR **(A)** or with GSR **(B)**. Top: the distribution of Fisher's Z across region pairs were averaged across the 25 subjects for either without scrubbing (blue) or with scrubbing (red). Bottom: The difference in distribution between with and without scrubbing was averaged across the 25 subjects. Scrubbing reduces the correlation coefficients overall in the case of without GSR, i.e., left shifting the distribution. However, in the case of with GSR, the mean-centered distribution (GSR's property) is widened by scrubbing, i.e., scrubbing increased the possibility of extreme correlation values.

The present work explored the interaction of GSR with a number of other approaches thought to remove the impact of nuisance signals, including motion—namely, scrubbing, partial correlation and standardization. In the case where data is processed without GSR, we found that scrubbing reduced the impact of motion more than any of the other individual-level correction strategies, though still appeared to be less effective than GSR alone (e.g., the mean correlation between motion and small-worldness across densities 6–44% for Friston 24 + scrubbing: *r* = −0.20; while for Friston 24 + GSR: *r* = 0.03). Importantly, scrubbing did not produce any of the alterations in hub ranking or other topological parameters that were seen with GSR at higher densities. Thus, the effect of scrubbing is qualitatively different from GSR. Furthermore, when scrubbing was combining with GSR, as recently recommended by Power et al. (2014), the decreases in motion-density relationships produced through GSR alone, were less profound—suggesting a performance decrease. This may be explained in part by the introduction of more extreme correlation values through scrubbing (Figure 11). As shown in our prior work (Yan et al., 2013a), this is to be expected, as scrubbing inherently decreases the degrees of freedom, and systematic differences can be introduced across subjects as a function of the number of frames scrubbed. As suggested by Power et al. (2014), one can try to balance the impact of scrubbing in group comparisons by balancing the number of frames scrubbed between groups, but this cannot be easily accomplished in the study of inter-individual differences.

Under no condition was partial correlation (using graphical lasso) able to remove motion relationships to the extent that GSR was able to. This was surprising, as a signal present throughout the brain due to motion should be accounted for by partial correlation. One possibility that will be discussed in the next section is that residual relationships with motion may reflect the neural correlates of head motion, which would be expected to survive correction with partial correlation. Regarding standardization approaches, we found that regression of mean iFC and mean FD at the connection and topologic parameter stages was effective in removing the majority of relationships with motion, but once again, not as completely as GSR. In sum, GSR appears to possess a unique property that clearly merits future understanding, even if the approach itself is not well justified for continued use by the literature.

### Motion artifact vs. motion-related neural activity

Our prior work raised a key concern in the interpretation of motion-relationships with the BOLD signal and its derivatives—namely the possibility that they may in part be driven by the neural origins of motion, or reflect kinetic traits, rather than being solely the product of intensity fluctuations induced by motion (Yan et al., 2013a). In our prior work, this notion was supported by findings that low and high motion framewise displacements had differential effects on the BOLD signal. For individuals with a high frequency of framewise displacements greater than 0.2 mm, we found negative motion-BOLD relationships in the prefrontal areas, where displacements resulting from head motions are greatest; for individuals with particularly high amounts of motion (e.g., children), these negative relationships were even more widespread throughout the brain. Scrubbing largely removed these negative relationships. In contrast, positive motion-BOLD relationships were primarily present in motor-related cortices (e.g., primary motor, supplementary motor) and were relatively unaffected by scrubbing procedures—suggesting against origins in imaging artifact. One other theme of note arose from our analysis of relationships between differences in motion and differences in R-fMRI metrics across participants. In these analyses, we found that individuals with higher motion tended to have higher scores for a number of R-fMRI measures in motor-related cortices, and lower in default mode regions.

In the present work, we note that individuals with higher motion appeared to be characterized by higher centrality in dorsal parietal and dorsal frontal areas, and lower centrality in the default network—a finding that remains after motion correction approaches, including scrubbing. While this could still be a reflection of problematic effects of the low degree of motion present in the data, we find this highly unlikely. Instead, we posit, that our findings may in fact reflect either a trait marker of individual with higher kinetic traits, or at least higher kinetic states during the scan session. The unique ability of GSR to remove motion relationships is interesting, as it demotes the centrality of those regions that appear to be most associated with motion (even in scrubbed data) and increase the centrality of regions least associated with motion (Figure 10). Given that the global signal is known to have neural components (Scholvinck et al., 2010), a link may exist. Nonetheless, future efforts may benefit from working to find novel (and likely multimodal) ways of differentiating between image artifacts resulting from head motion and motion-related neural activity.

### Emerging recommendations for optimizing processing for graph theoretical analysis: what to or not to do

While a growing number of studies have begun to revisit the challenges of motion-correction for the purposes of R-fMRI, significant empirical and analytic work is needed before developing guidelines for addressing motion. Nonetheless, the present work has yielded multiple insights to help guide researchers as follows:

- *Global Signal Regression.* GSR appears to be the single-most effective approach for reducing motion-relationships—both at the individual and group-level. Despite this seeming success, we cannot recommend continued use of GSR without caution, as it can alter relative relationships within the connectome graph in a way that is unexplainable at present (particularly when weaker connections are included in the graph—i.e., when higher density graphs are used). Neither aggressive motion-correction (i.e., scrubbing) nor statistically accepted methods to accounting for confounding global signals (i.e., partial correlation) altered hub relationships, as GSR did.
- *Individual-level Motion Correction.* Consistent with prior reports, we found that neither model-based nor volume censoring approaches to motion correction are adequate for the removal of motion-relationships completely at the individual level.
- *Group-level Motion Correction.* Consistent with prior reports, we found that group-level covariate analysis (e.g., ANCOVA) is beneficial as a means of alleviating confounding motion effects in the study of inter-individual or population differences in topological parameters (Fair et al., 2012; Satterthwaite et al., 2012; Van Dijk et al., 2012; Yan et al., 2013a). We found correction in the final stage of analysis to be more advantageous to earlier group-level correction prior to graph construction, and the combination of the two approaches without clear merit.
- *Partial Correlation.* Using partial correlation rather than full correlation can reduce head motion effects substantially, due to its ability to remove signals present throughout the connectivity matrix. Calculation of partial correlation via graphical lasso is an effective way of overcoming insufficient number of degrees of freedom present in most R-fMRI time series. However, further studies are needed to evaluate the residual motion effects, as well as neurobiological significance of the brain graphs revealed by graphical lasso rather than full correlation.

### Limitations

Several limitations in the current work merit consideration. First, the head motion parameters were estimated from the fMRI data themselves, and limited to between-volume motions (i.e., motion occurring within the period of a single scan volume cannot be accounted for). Future studies require objective external measurement of motion to obtain a true gold standard of head motion. Second, simultaneously recorded cardiac and respiratory signals were not available for the dataset used in the current study, which prevented the definitive separation of head motion effects from physiological noise sources as well as meaningful neural signals. Third, the current methods explored graphical lasso as a statistical method to evaluate partial correlation; although effective and generally accepted, alternative approaches exist (e.g., ridge and elastic net) and should be considered for further exploration. Fourth, in order to facilitate group comparisons, we created two groups of participants using mean FD (high motion vs. low motion) for our two-sample *t-test* based analyses; however, mean FD is not all encompassing—other aspect of motion attributes can vary across participants and groups in an uncontrolled manner. Additionally, while we controlled sex and age between the two groups, other uncontrolled traits (e.g., IQ, social economical status, extraverts vs. introverts) may differ between the two groups. Future studies may consider the creation of within-subject designs for comparison of motion states, i.e., high motion vs. low motion scans for each subject. Fifth, for the group-level mean FD correction, we only take mean FD itself but not the interaction term (mean FD ^*^ Group) into account. If the interaction term is modeled and significant, interpretation of findings related to the main group effect can be difficult. In such a case, methods such as the Johnson-Neyman procedure can be carried out to determine within which range of covariates the main group effect is significant, and which range is not (D'Alonzo, 2004). Finally, in our previous work on standardization (Yan et al., 2013b), we standardized global SD beyond global mean (e.g., method of mean regression + SD division). In the current work, SD division for each individual had no effects on the graph construction, as it doesn't change the relative order of connections for a given participant. Further studies focusing on addressing the multiplicative effects might be helpful in mitigating head motion effects.

## Conclusions

While graph theoretical measures, including local and global topological parameters, possess significant promise for the advancement of our quantification and understanding of inter-individual differences in human brain function, they can be profoundly confounded by the presence of motion if not properly accounted for. The present work explored various options to individual-level correction approaches, generating a set of recommendations for future work and demonstrating the continued necessity for using ANCOVA-based corrections at the group-level. A key challenge for the field as it moves forward is to develop empirical and analytic approaches that are capable of differentiating associations with motion between reflective of artifact and reflective of neural signals underlying motion in the scanner, or trait markers.

## Conflict of interest statement

The authors declare that the research was conducted in the absence of any commercial or financial relationships that could be construed as a potential conflict of interest.

## Footnotes

^1^In order to achieve densities as close as the desired range, the regularization penalties were varied from 0.0001 to 0.001 in a step of 0.0001, and then from 0.001 to 1 in a step of 0.001, and the penalty resulting in the density closest to the desired value was chosen. Consequently, the selected inverse covariance matrices are very close to the desired densities.

^2^We calculated the all voxel-to-voxel mean iFC as follows: (1) normalize the time courses of all the voxels to zero mean and unit variance; (2) calculate the mean signal across the brain (“global signal”); (3) calculate correlation between this “global signal” and all the other voxels (a simple dot product and then divided by *n* − 1); (4) calculate the mean value of the correlation coefficients across brain. This mean correlation coefficient is equivalent to the mean of all voxel-to-voxel correlations. This calculation is similar to the L^2^ norm method recently proposed by Saad et al. (2013), but in a more intuitive form. To improve the normality of such a value for the purpose of standardization, we converted the mean iFC into Fisher's *z* value.

## Appendix

**Table A1:** **Abbreviations for the regions in the AAL-atlas**.

**Cortical regions**	**Abbreviations**
Precental gyrus	PreCG
Superior frontal gyrus, dorsolateral	SFGdor
Superior frontal gyrus, orbital part	ORBsup
Middle frontal gyrus	MFG
Middle frontal gyrus, orbital part	ORBmid
Inferior frontal gyrus, opercular part	IFGoperc
Inferior frontal gyrus, triangular part	IFGtriang
Inferior frontal gyrus, orbital part	ORBinf
Rolandic operculum	ROL
Supplementary motor area	SMA
Olfactory cortex	OLF
Superior frontal gyrus, medial	SFGmed
Superior frontal gyrus, medial orbital	ORBsupmed
Gyrus rectus	REC
Insula	INS
Anterior cingulate and paracingulate gyri	ACG
Middle cingulate and paracingulate gyri	DCG
Posterior cingulate gyrus	PCG
Hippocampus	HIP
Parahippocampal gyrus	PHG
Amygdala	AMYG
Calcarine fissure and surrounding cortex	CAL
Cuneus	CUN
Lingual gyrus	LING
Superior occipital gyrus	SOG
Middle occipital gyrus	MOG
Inferior occipital gyrus	IOG
Fusiform gyrus	FFG
Postcentral gyrus	PoCG
Superior parietal gyrus	SPG
Inferior parietal, but supramarginal and angular gyri	IPL
Supramarginal gyrus	SMG
Angular gyrus	ANG
Precuneus	PCUN
Paracentral lobule	PCL
Caudate nucleus	CAU
Lenticular nucleus, putamen	PUT
Lenticular nucleus, pallidum	PAL
Thalamus	THA
Heschl gyrus	HES
Superior temporal gyrus	STG
Temporal pole: superior temporal gyrus	TPOsup
Middle temporal gyrus	MTG
Temporal pole: middle temporal gyrus	TPOmid
Inferior temporal gyrus	ITG
